# Editorial: Extracellular matrix dynamics in biology, bioengineering, and pathology, volume II

**DOI:** 10.3389/fcell.2022.1105566

**Published:** 2022-12-02

**Authors:** Rajprasad Loganathan, Hiromi Yanagisawa, Eileen Gentleman, Charles D. Little, Jeffrey A. Weiss

**Affiliations:** ^1^ Developmental and Integrative Systems Biology Lab, Department of Biological Sciences, Wichita State University, Wichita, KS, United States; ^2^ Life Science Center for Survival Dynamics, TARA, University of Tsukuba, Tsukuba, Japan; ^3^ Centre for Craniofacial and Regenerative Biology, King’s College London, London, United Kingdom; ^4^ Department of Cell Biology and Physiology, University of Kansas Medical Center, Kansas City, KS, United States; ^5^ Department of Biomedical Engineering, The University of Utah, Salt Lake City, UT, United States

**Keywords:** ECM, biology, bioengineering, pathology, network, dynamics

## Introduction

The current volume of articles focused on extracellular matrix (ECM) presents some recent developments in biology, bioengineering, and pathology that reiterate the central theme of our previous volume. Collectively, these articles provide multidisciplinary evidence that the ECM operates as a highly dynamic functional framework in physiology and pathology. Furthermore, they underscore the opportunities available for future explorations that can help develop more robust models of ECM structure and function for human health and disease.

## Biology

Petzold and Gentleman offer a comprehensive review of mechanical cues and their impact on stem cells and embryogenesis. Their article contrasts the effects of mechanotransduction from intrinsic cues vs. extrinsic cues for cell differentiation. ECM mechanical properties and topography take the central stage in their discussion of cellular response to mechanotransduction. The authors also provide a coherent summary of the bidirectional structural changes that occur in the cell and the ECM mediated by a robust cytoskeletal signaling network. Furthermore, their discussion extends to the mechanoregulation of embryogenesis and the novel methods that have emerged in this field for the measurement of embryonic stiffness.

Few models of tendon developmental biology are available. Moreover, models of tendon development that allow investigation of craniofacial muscles have yet to be explored. Korntner et al. test the capacity of chick embryos to offer a system for the study of craniofacial tendon development. Using the jaw-closing tendon of the musculus adductor mandibulate externus and the jaw-opening tendon of the musculus depressor mandibulae, they characterize the developmental trajectory of cell and ECM morphology. They discover that several markers implicated in limb tendon formation are also present in embryonic jaw tendon. Their morphological and molecular biological characterizations of the developmental program directing chick jaw muscle tendon assembly provide a basis for future modeling of the developmental program shaping human masticatory tendons.

The study by López-Mengual et al. illustrates the critical role played by mechanical cues in the developing nervous system. Using the mouse embryonic explant system for the study of slices of developing brain in Matrigel preparations, the authors complement the methods of classical developmental biology with atomic force microscopy and traction force microscopy measurements to demonstrate differential tissue stiffness of the developing brain and its immediate effects on Cajal-Retzius cell migration and ultimate effect on neocortical development.

## Bioengineering

Bioengineering researchers have long provided an ever-expanding toolkit of methods to investigate cell and ECM mechanobiology. In that tradition, Scholp et al. present their design of a custom force-bioreactor that uses fibroblast-seeded fibrin gels to allow microscopy-based documentation of force generation in response to drug treatment. Lu et al. describe their findings on the use of pulmonary visceral pleura (PVP) as a potential biomaterial for tissue repair and reconstruction. They test PVP processed from both swine and bovine lungs to determine its structural characteristics, mechanical properties, cytotoxicity, and biocompatibility for rat sciatic nerve and skin repair.

ECM structural organization has long remained a fertile ground for investigations from diverse scientific fields. An application of methods from condensed matter physics to detect changes in collagen fiber organization in colorectal cancer is tested by Despotović and Ćosić. Their work shows that the collagen fiber straightness quantified using theoretical methods as a measure of ECM remodeling can be a useful indicator of the early stages in the development of colorectal cancer.

## Pathology

Knutsen et al. report on the therapeutic effects of K_ATP_ channel opener Minoxidil for the treatment of individuals with William-Beuren Syndrome (WBS), which occurs as a result of loss of one copy of the elastin gene ultimately causing large artery vasculopathy. After confirming the similarities in pathophysiological features of elastin heterozygous null mice with those of children with WBS they demonstrate partial remedy of cardiovascular features in mice. Their results provide the necessary evidence to suggest consideration of Minoxidil therapy under strict cardiac size monitoring as an adjunct to surgical intervention for humans with WBS cardiovascular pathology.

In addition to gene loss targeting the ECM architecture, defects of the cardiovascular system are also caused by the effects of aging on the ECM morphology. In their mini-review, Mammoto et al. describe the ECM changes that occur in aging aorta with a particular focus on the fragmentation of elastic fibers and excessive deposition and crosslinking of collagens as major drivers of aging-induced aortic dysfunction. Cardiac pathology is the focus of yet another investigation published in this Research Topic. Studying a rodent model of heart hypoplasia induced by congenital diaphragmatic hernia, Watson et al. suggest that changes in environmental cues such as ECM stiffness and mechanical force may underlie the pathological progression of heart hypoplasia by affecting the balance between cardiomyocyte cell proliferation and maturation.

From experiments showing the unexpected effects of prophylactically administered drugs on ECM functional organization in a rat model of elbow injury and *in vitro* collagen gel assay, David et al. report that simvastatin may be a suitable prophylactic drug therapy for preventing or mitigating post-traumatic joint contracture.

In their study on the effects of ECM on nerve injury and regeneration, Bauch et al., using a cerebellar slice culture model of demyelination/remyelination, provide evidence for the inhibitory effects of tenascin-C and tenascin-R on remyelination. Collectively, their results show the relevance of oligodendrocyte physiology and ECM dynamics for gaining a better understanding of debilitating pathologies, e.g., multiple sclerosis.

## Summary

As co-editors of this Research Topic, we thoroughly enjoyed learning about the aforementioned recent developments in ECM dynamics. We hope the readers of this Research Topic would also feel the same way.

